# Investigation of Toughening Mechanism of Virgin Asphalt by Blending with Waste Battery Powder

**DOI:** 10.3390/gels12020117

**Published:** 2026-01-28

**Authors:** Chenze Fang, Xu Guo, Yuanzhao Chen, Hui Li, Naisheng Guo, Zhenxia Li, Zongyuan Wu, Jingyu Yang, Tengteng Guo

**Affiliations:** 1 School of Civil Engineering and Transportation, North China University of Water Resources and Electric Power, Zhengzhou 450045, China; fangchenze@126.com (C.F.); gx20032025@126.com (X.G.); zhenxiali2009@ncwu.edu.cn (Z.L.); wuzongyuan@ncwu.edu.cn (Z.W.); yangjingyu@ncwu.edu.cn (J.Y.); guotth@ncwu.edu.cn (T.G.); 2 Electric Power-Henan Provincial Institute of Transportation Planning and Design Co., Ltd., Green, Low Carbon, and High Performance Road Materials Research and Development Center, North China University of Water Resources, Zhengzhou 450045, China; 3 Technology Innovation Center of Henan Transport Industry of Utilization of Solid Waste Resources in Trafffc Engineering, North China University of Water Resources and Electric Power, Zhengzhou 450045, China; 4 Henan Province Engineering Technology Research Center of Environment Friendly and High-Performance Pavement Materials, Zhengzhou 450045, China; 5 Department of Transportation, Southeast University, Nanjing 210096, China; 6 Department of Transportation Engineering, Dalian Maritime University, Dalian 116026, China; naishengguo@126.com

**Keywords:** road engineering, waste battery powder, asphalt toughening, cracking area, damage variables, activation energy

## Abstract

Waste battery powder (WBP) can effectively enhance the service performance of virgin asphalt with sol–gel structures; however, its toughening mechanism for sol–gel virgin asphalt still lacks rigorous mechanical characterization. Therefore, the objective of this study is to investigate the toughening of WBP-modified asphalt based on the mechanical parameter of cracking area. First, a 12% content of WBP was incorporated into the sol–gel 70# virgin asphalt to prepare WBP-modified asphalt and its fatigue performance was evaluated through linear amplitude, non-damage, and damage time sweep tests. Then, energy–mechanics balance equations were used to establish a cracking area model. Furthermore, the asphalt cracking area was employed to quantify its induced damage and determine the representative rate for the cracking damage process (*k*_cd_). Finally, the activation energy for cracking damage (*E*_acd_) was used to quantify the difficulty of the cracking damage process. The scanning electron microscope test was employed to examine the microstructure of WBP-modified asphalt and the *E*_acd_ and microscopic morphology of WBP-modified asphalt were analyzed to reveal the toughening effect of WBP on virgin asphalt. The results showed that WBP-modified asphalt exhibits three nonlinear cracking stages, with a lower cracking rate than virgin asphalt. Its cracking damage generally increases over time, and the damage evolution parameter *β* serves as *k*_cd_. The micro-grooves and wrinkles of WBP improve bonding to asphalt, increasing the *E*_acd_ of sol–gel 70# virgin asphalt from 10.6 to 23.88 kJ·mol^−1^, thus achieving toughening. In summary, the fatigue damage process of WBP-modified asphalt can be characterized by the kinetic parameters *β* and *E*_acd_.

## 1. Introduction

The rapid development of new energy battery technology has significantly improved people’s living standards. However, the issue of discarded batteries as household waste has increasingly troubled people’s daily lives. According to incomplete statistics, over 95% of used batteries globally are treated as solid waste through incineration and landfill disposal, resulting in significant pollution to watershed soils, groundwater, and the ecological environment in 2024 [1,2,3]. Scientifically applying waste battery resources to transportation infrastructure construction can effectively promote the high-value utilization of waste battery resources and the development of low-carbon building material technologies.

According to classical colloid theory, conventional virgin asphalt without modified additives typically exhibits characteristic sol–gel structural features, which ensure its excellent high-temperature stability and low-temperature crack resistance. Its large-scale production and application in road engineering can reasonably be regarded as a key approach for the treatment and utilization of waste batteries [4,5,6]. The enhancement of asphalt material performance by the components of waste battery materials has been confirmed by many scholars. In general, the related asphalt modifiers can be categorized into two types. The corresponding first type of asphalt modifier is carbon materials [7,8,9]. Jiang et al. [10] described high-temperature pyrolysis and demonstrated that the high-temperature pyrolysis of waste tires can produce high-quality carbon black. Feng et al. [11] investigated the preparation process of the conductive asphalt mixture and proved that carbon fiber added to the mixture can reduce its deformation under loading. He et al. [12,13,14] evaluated the road performance of graphene-modified asphalt and the results showed that graphene can significantly improve the high-temperature stability of asphalt. However, as a modifier, graphene is prone to agglomeration and has a high manufacturing cost. The corresponding second type of asphalt modifier is metal oxide materials [15,16,17]. Zhu et al. [18] found that manganese dioxide improved the microwave heating performance more effectively than magnetite powder. Zhao et al. [19,20] developed a novel approach for value-added recycling of wood waste by constructing porous carbon structures (Pc-S) on aggregate surfaces. Compared to basalt materials, the carbon-modified granite produced via this method exhibits enhanced interfacial bonding properties, resulting in an over 78.9% improvement in resistance to water ingress. In summary, asphalt performance can be enhanced by the carbon materials and metal oxides in waste battery powder.

The above studies have extracted single-component materials from waste batteries with complex compositions for preparing modified asphalt, which is a complicated process [21,22,23] and obviously not easy to realize. Therefore, Fan [24] used the whole waste battery powder (WBP) as a modifier and verified the feasibility of preparing modified asphalt using whole waste battery powder. Based on rheological performance analysis, the optimal addition of the WBP was recommended to be 9%. Meng et al. [25,26] demonstrated that asphalt can exhibit a significant encapsulation effect on heavy metals in the WBP, effectively preventing pollution caused by the leaching of heavy metals. Meng et al. [27] employed microscopic techniques, including FTIR and SEM, and found that the carbon in WBP exhibited relatively high crystallinity and content. The incorporation of WBP enhanced the bond energy of the methylene groups, thereby strengthening the molecular interactions between the asphalt and WBP and consequently improving the pavement performance of the modified asphalt. In addition, the grooves and wrinkles observed on the microscopic surface of WBP were analyzed and confirmed to enhance the bonding strength between aggregates and asphalt effectively. In summary, WBP can be used as a safe and effective asphalt modifier.

Although the aforementioned studies have demonstrated that asphalt materials can serve as an effective carrier for recycling waste batteries, most of the related studies have focused on the effect of waste battery powder materials on the asphalt’s rheological behavior, particularly lacking systematic mechanical characterization of the cracking damage behavior of the asphalt modified by waste battery powder materials under cyclic loading. Furthermore, the damage variable definition methods based on traditional phenomenological indicators (such as modulus and strength attenuation) lack rigorous mechanical derivation [28], making it difficult to characterize the significant differences in mechanical response between damaged and undamaged asphalt, and challenging to accurately define the damage variables of waste battery material-modified asphalt. This results in a lack of scientifically rigorous mechanical explanations for the toughening mechanism of the WBP on virgin asphalt.

At the same time, the existing studies [29] indicate that the energy–mechanics method can accurately describe the damage behavior of asphalt in terms of its mechanical nature by establishing energy–mechanics equilibrium equations for damaged and undamaged asphalt. Given this, the objective of this study is to utilize the energy–mechanics method to derive a cracking area indicator with clear mechanical significance, and to investigate the toughening mechanism of the WBP on sol–gel virgin asphalt based on the cracking area. The significant contribution of this study lies in establishing a cracking-area-based damage model derived from energy–mechanical balance equations and further proposing a damage activation energy evaluation method based on kinetics theory, which provides a theoretical basis for extending the service life of asphalt pavements by utilizing waste battery resources. First, the energy–mechanics method was used to establish the cracking area model of WBP-modified asphalt for analyzing its cracking area response laws. Then, a damage model for the WBP-modified asphalt was developed to determine its representative rate for the cracking damage process (*k*_cd_). Finally, the activation energy for cracking damage (*E*_acd_) was used to quantify the difficulty of the cracking damage process, and the WBP’s microscopic morphology and *E*_acd_ were utilized to analyze the toughening mechanism of WBP on sol–gel virgin asphalt.

## 2. Results and Discussion

### 2.1. Cracking Area Analysis of WBP-Modified Asphalt Based on Energy–Mechanics Method

#### 2.1.1. Establishment of Asphalt Cracking Area Model Based on Energy–Mechanics Method

**Figure 1:**
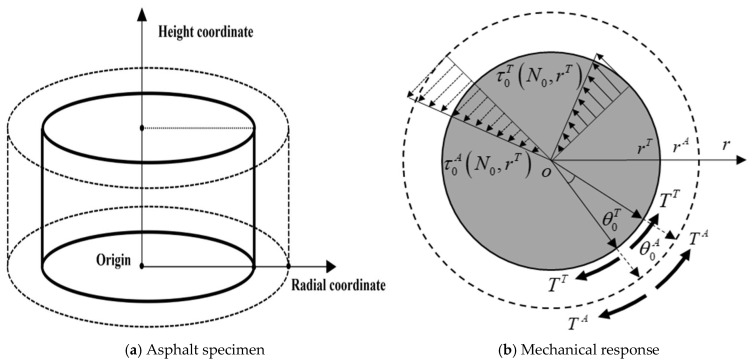
Schematic diagrams of asphalt specimen and its mechanical response during cyclic loading process.

Figure 1 illustrates the asphalt specimen and its force distribution under cyclic shear loading. As shown in Figure 1a, the entire asphalt specimen (cylinder indicated by dashed lines) mainly consists of the crack-damaged asphalt section (annular region indicated by dashed and solid lines) and intact asphalt section (cylinder formed by solid lines) [30]. During loading, the crack-damaged asphalt propagates progressively from the outer periphery of the specimen toward its center.

The entire asphalt specimen and intact asphalt specimen are, respectively, referred to as the apparent asphalt and true asphalt and their mechanical responses are shown in Figure 1b. For the apparent asphalt during the cyclic shearing process, the stress and strain are shown in Equations (1) and (2).

(1) $$\tau^{A}\left(t,r\right)=\tau_{0}^{A}\left(t_{0},r\right)\sin\left(\omega t\right)$$

(2) $$\gamma^{A}\left(t,r\right)=\gamma_{0}^{A}\left(t_{0},r\right)\sin\left(\omega t-\delta \right)$$

where *τ^A^*(*t*,*r*) and *γ^A^*(*t*,*r*) represent the stress and strain induced in the apparent asphalt, respectively; *t* denotes loading time; *r* denotes specimen radius length; *t*_0_ denotes the initial time; $\tau_{0}^{A}\left(t_{0},r\right)$ and $\gamma_{0}^{A}\left(t_{0},r\right)$ denote shear stress amplitude and shear strain amplitude, respectively; *ω* denotes angular velocity; and *δ* denotes phase angle.

For the true asphalt during the cyclic shearing process, its stress and strain are expressed by Equations (3) and (4).

(3) $$\tau^{T}\left(t,r\right)=\tau_{0}^{T}\left(t_{0},r\right)\sin\left(\omega t\right)$$

(4) $$\gamma^{T}\left(t,r\right)=\gamma_{0}^{T}\left(t_{0},r\right)\sin\left(\omega t-\delta \right)$$

where *τ^T^*(*t*,*r*) and $\tau_{0}^{T}\left(t_{0},r\right)$ denote the shear stress and its amplitude in true asphalt, respectively; *γ^T^*(*t*,*r*) and $\gamma_{0}^{T}\left(t_{0},r\right)$ denote the shear strain and its amplitude in true asphalt, respectively.

The energy–mechanics approach for establishing a cracking area model relies on two fundamental assumptions: First, the apparent asphalt consists of cracked asphalt and true asphalt. Second, an equilibrium exists between the true asphalt and apparent asphalt in terms of recoverable strain energy, dissipated strain energy, and torque. Although the crack-damaged asphalt can lead to significant differences in the mechanical response between true asphalt and apparent asphalt, relevant studies [26,27,28] indicate that the two exhibit energy–mechanical equilibrium equations in terms of recoverable strain energy, dissipated strain energy, and torque, as shown in Equations (5)–(7).

(5) $$RSE^{T}=RSE^{A}$$

(6) $$DSE^{T}=DSE^{A}$$

(7) $$T^{T}=T^{A}$$

where *RSE^T^* and *RSE^A^* represent the recoverable strain energy of true asphalt and apparent asphalt, respectively; *DSE^T^* and *DSE^A^* represent the dissipated strain energy of true asphalt and apparent asphalt, respectively; *T^T^* and *T^A^* represent the torque of true asphalt and apparent asphalt, respectively.

By combining the three energy–mechanical equilibrium equations above, a cracking area model for asphalt can be derived, as shown in Equation (8). Equation (8) indicates that modulus decay can quantitatively reflect the cracking area of asphalt. Since the cracking area provides an intuitive quantification of asphalt damage, this study will analyze the damage evolution of asphalt during repeated loading based on this cracking area model.

(8) $$CA=\pi {\left(r^{A}\right)}^{2}\left\{1-\left[1-{\left(1-G_{r}^{0.25}\right)}^{2}\right]\right\}$$

where *CA* is the cracking area of the asphalt; *r^A^* is the apparent asphalt radius, numerically equivalent to the radius of the entire asphalt specimen; *G_r_* is the modulus ratio, numerically equal to the shear modulus corresponding to the apparent asphalt divided by that corresponding to the true asphalt.

**Figure 2:**
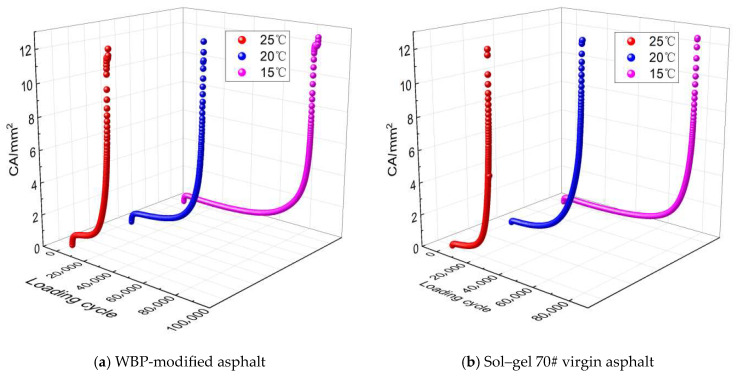
Cracking area curves of tested asphalt.

According to the derived cracking area model shown in Equation (8), the cracking area results for WBP-modified and sol–gel 70# virgin asphalt are obtained, as depicted in Figure 2. It should be noted that the research subject, WBP-modified asphalt, in this paper refers to modified asphalt containing 12% waste battery powder, with its detailed preparation process described in Section 4.1.2. As observed in Figure 2, the cracking area curves of WBP-modified and sol–gel 70# virgin asphalt induced by cyclic loading exhibits the similar nonlinear cumulative trend under the intermediate temperatures of 15 °C, 20 °C, and 25 °C. A straightforward comparison between Figure 2a,b shows that, under identical temperature and stress conditions, WBP-modified asphalt exhibits a slower overall trend in crack area growth compared to virgin asphalt. This is particularly evident during the mid-to-late loading stages (corresponding to the second and third phases), where the crack area curve of the virgin asphalt remains below that of the virgin asphalt, indicating that the incorporation of waste battery powder can delay crack propagation.

#### 2.1.2. Cracking Area Evolution Analysis of WBP-Modified Asphalt

**Figure 3:**
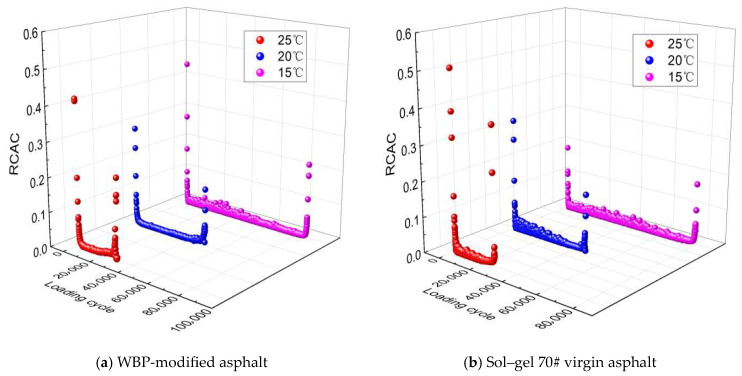
Curve results of cracking area and rate of cracking area change for WBP-modified asphalt.

To quantitatively analyze the evolution laws of cracking area in the WBP-modified asphalt, the rate of cracking area change (*RCAC*) is defined as shown in Equation (9). Based on Equations (8) and (9), the curve results of the cracking area and rate of cracking area change for WBP-modified and sol–gel 70# virgin asphalt are obtained, as shown in Figure 3.

(9) $$RCAC=\frac{\left|CA_{b}-CA_{a}\right|}{CA_{a}\left(b-a\right)}$$

where *RCAC* is rate of cracking area change; *CA_a_* is cracking area of the asphalt in the *a-*th cycle; *CA_b_* is the cracking area of the asphalt in the *b-*th cycle.

As observed in Figure 3, the cracking area response of the WBP-modified and sol–gel 70# virgin asphalt can be divided into the first phase of sudden and rapid accumulation, the second phase of long-term constant speed fluctuation, and the third phase with a brief and rapid leap. During the first phase, the cracking area rapidly accumulates from 0 to a certain level of cracking area, but the rate of cracking area change gradually decreases. After the cracking area evolution enters the second phase, the cracking area continues to maintain a stable cumulative trend and the rate of cracking area change remains generally stable with slight fluctuations. At this phase, the WBP-modified and sol–gel 70# virgin asphalt exhibit stable crack resistance. During the final or third phase, the cracking area sharply accumulates and the rate of cracking area change rapidly increases. Finally, the WBP-modified and sol–gel 70# virgin asphalt nearly completely lose their cracking resistance and experience fatigue failure. The analysis above indicates that the rate of cracking area change can reflect the cracking resistance of the WBP-modified and sol–gel 70# virgin asphalt and that the two exhibit a negative correlation. Therefore, this study defines the loading cycle at the tangent intersection point corresponding to the second and third phases of the rate of cracking area change curves as the fatigue life. Compared to the WBP-modified asphalt, the RCAC curve of virgin asphalt enters the steeply rising third stage earlier. This phenomenon indicates that under identical cyclic loading, virgin asphalt loses its fatigue crack resistance more rapidly, thereby reaching fatigue failure sooner. In contrast, modified asphalt incorporating WBP delays the transition to the failure stage, exhibiting more enduring and stable crack resistance. This directly confirms that WBP incorporation effectively enhances the fatigue crack resistance of virgin asphalt.

The fatigue life results obtained through analysis of the rate of cracking area change are shown in Table 1. As indicated in Table 1, when the temperature–loading conditions are 15 °C–250 kPa, 20 °C–100 kPa, and 25 °C–80 kPa, the obtained fatigue life results corresponding to the sol–gel 70# virgin asphalt are 79,530, 45,140, and 25,890, respectively, which correspond to increased values for the WBP-modified asphalt of 87,880, 50,466, and 29,022, respectively. The average increase in asphalt fatigue life by waste battery powder is 11.5%, which indicates that waste battery powder added to sol–gel 70# virgin asphalt can delay the cracking process of asphalt and improve its service life. The above analysis indicates the waste battery powder exhibits a toughening effect on virgin asphalt, which is in good agreement with the results in references [24,25,26,27].

### 2.2. Analysis of Representative Rate of Cracking Damage Process for WBP-Modified Asphalt

Compared to indirect indicators of asphalt damage, such as modulus decay and energy dissipation, the cracking area exhibits clear physical significance and quantifies the extent of asphalt damage. Therefore, this study defines the damage variable of WBP-modified and sol–gel 70# virgin asphalt as the ratio of the cracking area to the intact specimen area, as shown in Equation (10). It should be noted that damage variable definition methods based on traditional phenomenological indicators (such as modulus and strength attenuation) represent an indirect approach to quantifying damage, lacking rigorous mechanical derivation and a clear mechanical meaning. Damage definition methods based on crack area can directly quantify damage, thereby partially addressing these two shortcomings.

(10) $$D=\frac{CA_{N}}{SA}$$

where *D* is the damage variable; *CA_N_* is the specimen cracking area of the *N*-th cycle; *SA* is the intact area of the asphalt specimen, with a geometric size of r (radius) in this study, *SA* = *π*(*r^A^*)^2^.

**Figure 4:**
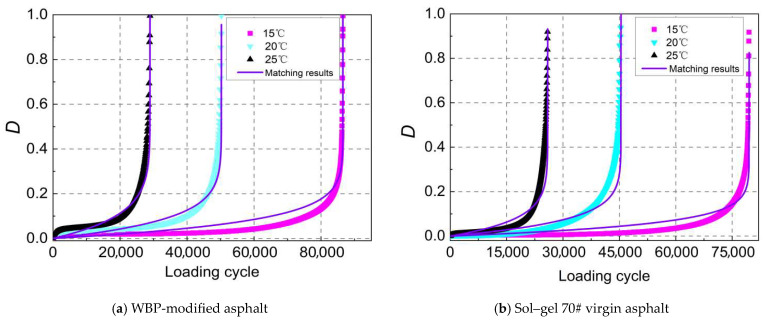
Fatigue damage curves of WBP-modified and sol–gel 70# virgin asphalt.

The determined damage curve of WBP-modified and sol–gel 70# virgin asphalt using Equation (10) is shown in Figure 4. As observed in Figure 4, the damage of both WBP-modified and sol–gel 70# virgin asphalt produced by cyclic shear exhibits an overall evolution trend with increasing rate. The existing research indicates that dissipated energy evolution can be used to quantitatively describe asphalt damage evolution, as shown below:

(11) $$\frac{dD}{dN}={\left(\frac{\partial W_{N}}{\partial N}\right)}^{\beta }$$

where $\frac{dD}{dN}$ is the damage evolution; $\frac{\partial W_{N}}{\partial_{N}}$ is the dissipated energy evolution; *β* is a material characteristic parameter that reflects the sensitivity of asphalt damage evolution to dissipated energy evolution. It should be noted that *β* quantifies the rate correlation between damage evolution and dissipated energy evolution: a larger *β* indicates that the asphalt damage process is more sensitive to the change of dissipated energy (i.e., a small change in dissipated energy will lead to a significant acceleration of damage); conversely, a smaller *β* means the damage evolution is less dependent on dissipated energy variation.

According to Equation (11), a damage model suitable for asphalt materials under cyclic loading can be derived, as shown in Equation (12) [31,32,33,34]. This damage model is used to match the damage curves of WBP-modified and sol–gel 70# virgin asphalt. The determined matching results are presented in Table 2 and Figure 4. As presented, the damage model expressed in Equation (12) can accurately capture the cracking damage process of WBP-modified and sol–gel 70# virgin asphalt under cyclic loading. Therefore, such a damage model will be used to quantitatively analyze the cracking damage process of WBP-modified and sol–gel 70# virgin asphalt.

(12) $$D=1-{\left(1-\frac{N}{N_{f}}\right)}^{\frac{1}{1+2\beta }}$$

*N_f_* refers to the total number of cyclic loading cycles when the asphalt specimen reaches the failure state.

The aforementioned cracking damage process is a complex rate-variable physical reaction. To quantify the corresponding reaction rate, taking the logarithm of Equation (13) yields the damage evolution equation in logarithmic coordinates, as shown below:

(13) $$\ln\left(\frac{dD}{dN}\right)=\beta \ln\left(\frac{\partial W_{N}}{\partial N}\right)$$

Equation (13) reveals that $\ln\left(\frac{dD}{dN}\right)$ is linearly correlated with $\ln\left(\frac{\partial W_{N}}{\partial N}\right)$ and the corresponding slope is equal to *β*. This *β* can reflect the damage evolution rate of the material under specific operating conditions. As the fatigue life fraction increases from 0 to 1, damage gradually evolves toward the failure threshold. During this process, the damage results are output by $D=1-{\left(1-\frac{N}{N_{f}}\right)}^{\frac{1}{1+2\beta }}$ and are directly reflected by the *β* [30,31,32]. As illustrated in Table 3 and Table 4, as the test temperature is sequentially decreased from 25 °C to 20 °C and then to 15 °C, both the *β* and *N_f_* values for the WBP-modified and sol–gel 70# virgin asphalt exhibit an increasing trend. This indicates that lowering the temperature can delay the propagation rate of asphalt fatigue cracks and the overall rate for the cracking damage process of WBP-modified and sol–gel 70# virgin asphalt is positively correlated with *β*.

### 2.3. Cracking Damage Activation Energy Analysis of WBP-Modified Asphalt

The representative rates for physical reaction processes at different temperatures can be accurately described by the *Arrhenius* kinetics equation, as shown in Equation (14) [35].

(14) $$k=Ae^{\frac{-E_{a}}{RT}}$$

where *k* is the representative rate; *E*_a_ is the activation energy; *A* is a pre-exponential parameter factor; *T* is the absolute temperature; and *R* is a gas constant.

For the damage model shown in Equation (12), *β* can be utilized as the representative rate for asphalt cracking damage. By combining Equations (12) and (14), the kinetics model for asphalt cracking damage is obtained:

(15) $$D=1-{\left(1-\frac{N}{N_{f}}\right)}^{\frac{1}{1+2Ae^{\frac{-E_{a}}{RT}}}}$$

where *E*_a_ represents activation energy for cracking damage.

According to kinetics theory [35], the activation energy for cracking damage represents the minimum energy required for cracking damage to occur in asphalt. The higher its value, the higher the energy threshold for asphalt cracking damage, meaning the asphalt cracking process is less likely to occur. Therefore, the toughening effect of waste battery powder on sol–gel virgin asphalt can be characterized by comparing the activation energy differences between the WBP-modified and sol–gel 70# virgin asphalt during cracking damage.

**Figure 5:**
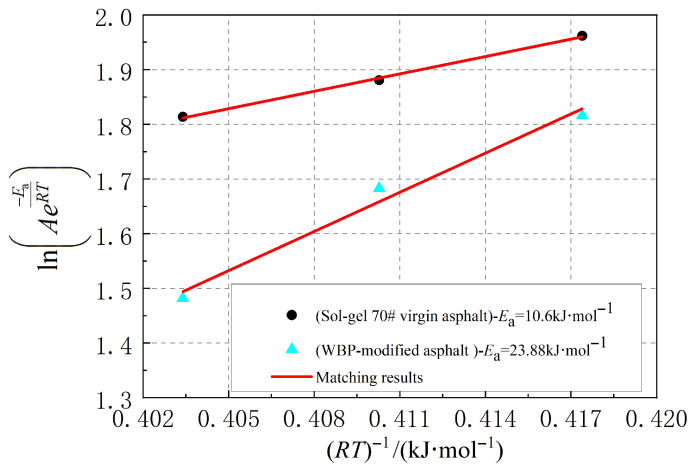
Representative rate for asphalt cracking damage.

To accurately obtain the activation energy for cracking damage in WBP-modified and sol–gel 70# virgin asphalt, the *β* at different temperatures were substituted into the *Arrhenius* kinetics equation in a double-logarithmic coordinate system as shown in Equation (16). The corresponding *β* were matched by Equation (16), as illustrated in Figure 5. It can be observed that the *Arrhenius* kinetics equation can accurately match the representative rates for the cracking damage of WBP-modified and sol–gel 70# virgin asphalt, further validating its effectiveness in quantifying the activation energy for cracking damage in WBP-modified and sol–gel 70# virgin asphalt. Taking the absolute value of the slope from the straight line in Figure 5 as the activation energy for cracking damage yields the results shown in Figure 5.

(16) $$\ln\left(Ae^{\frac{-E_{a}}{RT}}\right)=\ln A-\frac{E_{a}}{RT}$$

**Figure 6:**
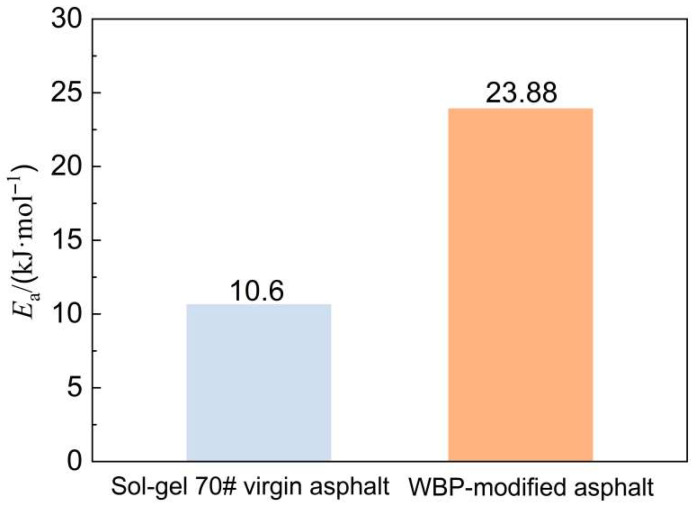
Activation energy for cracking damage.

As shown in Figure 6, the activation energy for cracking damage of sol–gel 70# virgin asphalt is 10.6 kJ·mol^−1^, while that of the WBP-modified asphalt is 23.88 kJ·mol^−1^. This indicates that the WBP-modified asphalt requires 13.28 kJ·mol^−1^ more energy than sol–gel 70# virgin asphalt to induce cracking damage. Thus, incorporating an appropriate amount of waste battery powder into sol–gel 70# virgin asphalt increases the difficulty of inducing cracking damage and extends its service life. Analysis of the activation energy for cracking damage confirms that waste battery powder exhibits a toughening effect on virgin asphalt. Such results occur mainly because the microstructure of waste battery powder can increase the difficulty of asphalt cracking under cyclic loading by improving the bonding ability between the asphalt and waste battery powder.

### 2.4. Microscopic Morphology Analysis of Waste Battery Powder and WBP-Modified Asphalt

**Figure 7:**
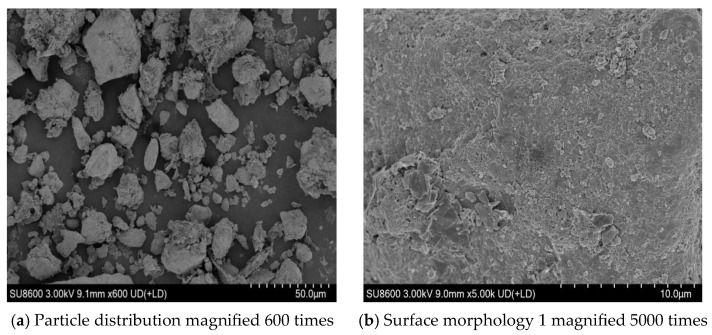

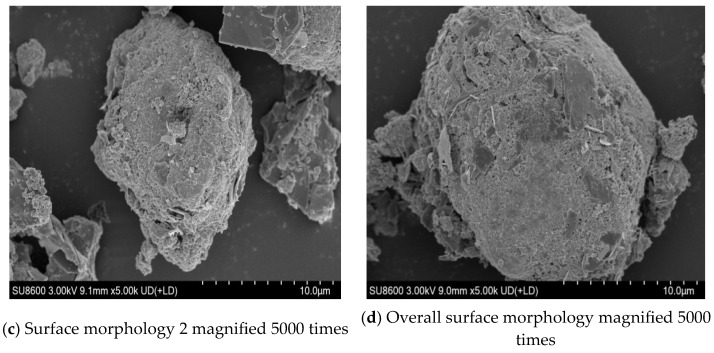
Microscopic morphology of waste battery powder.

The microstructure of waste battery powder obtained from the SEM test is illustrated in Figure 7a–d. Figure 7a shows the relatively uniform spatial distribution and size level of waste battery powder particles, which can effectively ensure the uniform distribution of waste battery powder in asphalt. The varying degrees of wrinkles or grooves distributed across the waste battery powder surface are shown in Figure 7b–d. Such rough surfaces can significantly increase the specific surface area of the waste battery powder, indicating a large bonding area between the asphalt and waste battery powder.

**Figure 8:**
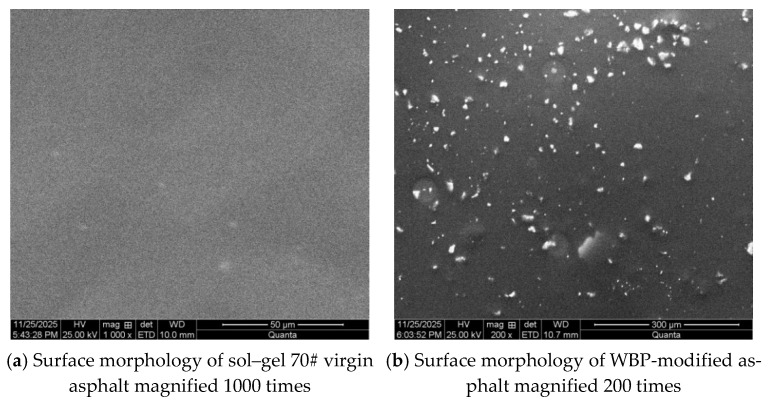

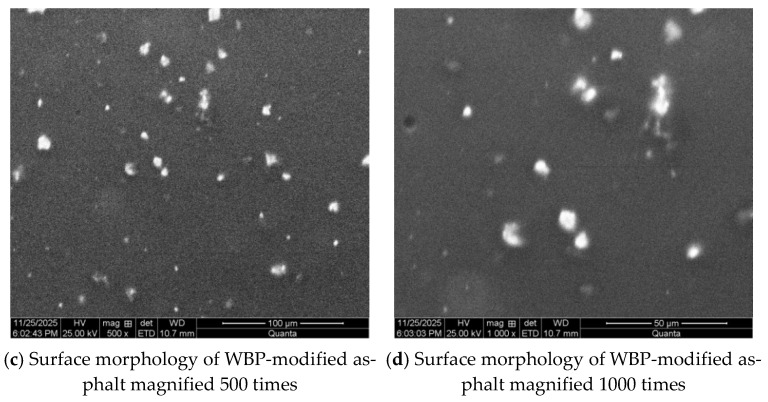
Microscopic morphology of WBP-modified and sol–gel 70# virgin asphalt.

Figure 8 shows the microstructure of the tested WBP-modified and sol–gel 70# virgin asphalt. As presented in Figure 8, the added waste battery powder is distributed relatively evenly throughout the sol–gel 70# virgin asphalt, which can enhance the intermolecular forces between the waste battery powder and asphalt and improve the corresponding adhesion. When the waste battery powder is used to modify the sol–gel 70# asphalt at temperatures exceeding 100 °C, the asphalt adopts a sol–gel structure predominantly in the sol state. Its grooves and folds adsorb asphalt, while some asphalt flows into its pores. Subsequently, as the asphalt cools from high temperatures to 15 °C room temperature, the sol–gel structure undergoes a transformation, gradually shifting toward a predominantly gel-state sol–gel structure. This gel hardening produces an anchoring effect, thereby enhancing the asphalt’s crack resistance under loading. In addition, existing research [27] indicates that the carbon in WBP exhibits relatively high crystallinity and content. The incorporation of WBP enhances the bond energy of methylene groups, thereby strengthening the molecular interactions between asphalt and WBP, and consequently improving the asphalt performance. The above microscopic analysis results are consistent with the analysis of activation energy for cracking damage, which validates the effectiveness of the analysis method proposed in this study for the toughening of waste battery powder on sol–gel 70# virgin asphalt.

## 3. Conclusions

The significant contribution of this study lies in establishing a cracking area-based damage model derived from energy–mechanics balance equations, which enables accurate quantification and characterization of the fatigue damage behavior of the WBP-modified asphalt under cyclic loading. In addition, a damage activation energy evaluation method based on kinetics theory is proposed, which is expected to provide a new theoretical basis for the quantitative analysis of toughening mechanisms and performance optimization of the WBP-modified asphalt materials. The main conclusions are summarized as follows:

(1) The cracking area accurately characterizes the fatigue damage of the WBP-modified asphalt, which exhibits an overall trend of rate-increased evolution under stress-controlled conditions.

(2) The pronounced nonlinear characteristics of fatigue damage in the WBP-modified asphalt result in a variable-rate physical reaction process during asphalt fatigue damage. The parameter *β* in the damage evolution equation serves as a representative rate, quantifying the overall speed of the asphalt fatigue damage process.

(3) The kinetics equation can characterize the variable-rate fatigue damage process of the WBP-modified asphalt by correlating representative fatigue damage rates. The Arrhenius kinetics equation becomes a linear equation in logarithmic coordinates, where the absolute value of the slope equals the activation energy for fatigue damage.

(4) The fatigue damage activation energy characterizes the minimum energy required for asphalt to sustain fatigue damage. A higher value indicates greater energy needed for fatigue damage to occur, signifying that the material is more resistant to fatigue damage. The micro-grooves and wrinkles on the surface of WBP modifiers increase the activation energy for fatigue damage, thereby improving the fatigue performance of the WBP-modified asphalt.

A method for analyzing the toughening effect of WBP on sol–gel virgin asphalt is proposed in this paper. The authors exclusively utilized 5# household mercury-free alkaline batteries and sol–gel 70# virgin asphalt as materials for preparing the WBP-modified asphalt. However, there are various types of waste batteries. Therefore, future research should validate the applicability of this method to other battery types. Subsequent studies will investigate the impact of different degrees of aging on the fatigue life of WBP-modified asphalt. Additionally, it is necessary to establish an intelligent decision-making system based on more extensive experimental data to determine the theory and technology for modified asphalt that takes into account factors such as environmental protection, performance, and economics.

## 4. Materials and Methods

### 4.1. Materials and Specimen Fabrication

#### 4.1.1. Sol–Gel Virgin Asphalt

The 70# virgin asphalt, provided by Shandong High-speed Construction Materials Co., Ltd. (Jinan, Shandong Province, China). Based on classical colloid theory, the conventional 70# virgin asphalt typically demonstrates characteristic sol–gel structural features. At temperatures above 100 °C, it exhibits a sol–gel structure dominated by the sol state. As the temperature gradually decreases from 100 °C to 50 °C, the sol–gel structure undergoes a progressive transformation. When the temperature falls below 50 °C, the 70# virgin asphalt exhibits a sol–gel structure where the gel state becomes more pronounced than the sol state. Such sol–gel structural features can enable 70# virgin asphalt binders to maintain stable viscoelastic properties across a wide temperature range. Sol–gel 70# virgin asphalt has been widely applied in road engineering. Therefore, the sol–gel 70# virgin asphalt was used as the virgin asphalt for the preparation of the WBP-modified asphalt, and Table 3 presents its basic indicators.

#### 4.1.2. Preparation Process of WBP-Modified Asphalt

According to the method in the literature of [15,24,25,26,27], the WBP was extracted from the recycled 5# household mercury-free alkaline batteries. First, the battery shell is removed, and the cathode material is ground into smaller particles using a grinder. Then, the battery particles are placed into the grinder for 20 min of grinding treatment, followed by screening with a 0.075 mm sieve to ensure a uniform particle size distribution of the waste battery powder. Finally, the screened WBP is placed into a 160 °C oven for 2 h to remove residual moisture and volatile impurities, ultimately yielding the final WBP. Table 4 shows the physical parameter indicators of the WBP.

**Figure 9:**
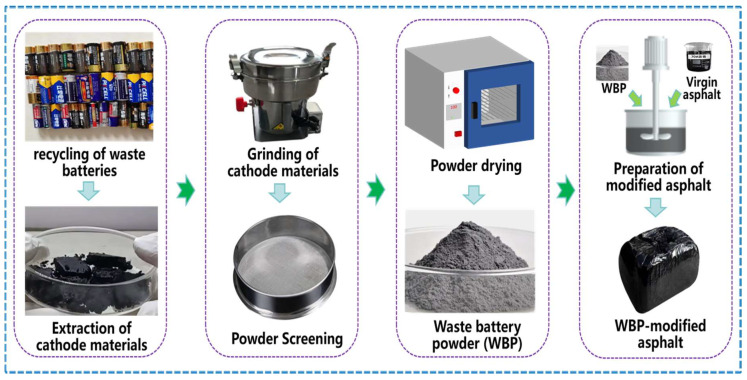
Recycling waste batteries for preparing WBP-modified asphalt.

According to the literature [24,27], the method of recycling waste batteries for preparing the WBP-modified asphalt can be described by Figure 9 and the optimal WBP content (by mass fraction) was found to be 12% through rheological performance testing. Therefore, in this study, the WBP content is set at 12% to prepare the WBP-modified asphalt according to the process. First, the 70 #VA is heated in an oven set to 165 °C until it reaches a molten state. Then, 12% by mass of WBP is added to the sol–gel 70# virgin asphalt and the mixture is sheared at 5000 r/min for 1 h at 155 °C. Next, the mixture is sheared at 1000 r/min for 10 min to remove any bubbles. Next, the asphalt is swollen and developed in an oven at 160 °C for one hour, at which point it adopts a sol–gel structure predominantly in the sol state. Finally, the asphalt is cooled to approximately 15 °C to produce the WBP-modified asphalt. At this point, the asphalt exhibits a sol–gel structure where the gel state is more pronounced than the sol state.

#### 4.1.3. Specimen Fabrication

**Figure 10:**
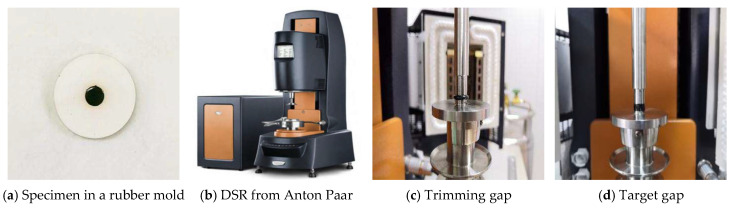
Preparation of 70# Virgin Asphalt and WBP Modified Asphalt Samples.

A dynamic shear rheometer (DSR) was selected as the molding and loading equipment for the sol–gel 70# virgin asphalt and the WBP-modified asphalt.. The DSR selected in this paper was produced by Tianjin Gangyuan test instrument factory (Tianjin, China). First, the hot flowing asphalt is placed into a rubber mold for fabricating a cylindrical asphalt specimen. Then, the cylindrical specimen is placed into the DSR to form the final test specimens. The trimming gap and target gap of DSR are set as 2050 μm and 2000 μm, respectively. The molding diameter and height are set as 8 mm and 2 mm for fabricating the cylindrical specimens of the 70# virgin and the WBP-modified asphalt, as shown in Figure 10

### 4.2. Test Methods

In this study, 10 Hz was selected as the loading frequency for the conducted tests of the WBP-modified and sol–gel 70# virgin asphalt, with five parallel tests set up. Existing studies [36,37] have shown that asphalt fatigue cracking mainly occurs in the 15–25 °C intermediate temperature range, and related studies often conduct fatigue tests under intermediate temperature conditions. Therefore, the asphalt fatigue test temperatures selected for this study are 15 °C, 20 °C, and 25 °C. At these temperatures, the tested asphalt exhibits a sol–gel structure where the gel state is more pronounced than the sol state.

#### 4.2.1. Linear Amplitude Time Sweep (LATS) Test

**Figure 11:**
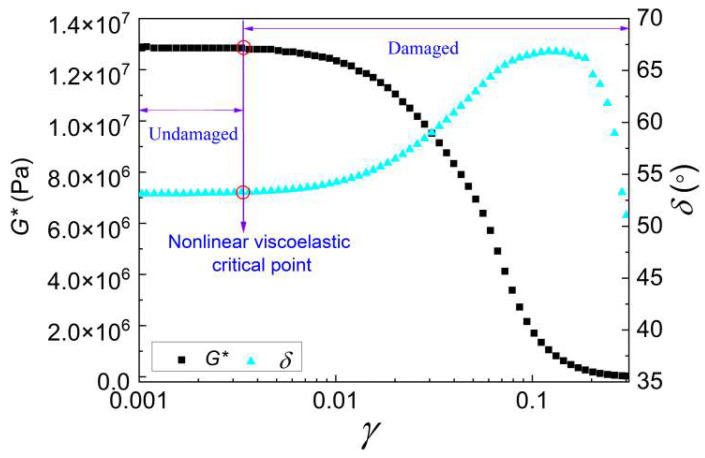
LATS test results of WBP-modified asphalt.

The LATS tests were performed for quantifying the nonlinear viscoelastic critical shear stress (*τ*_cri_). The applied loading control mode is a strain-controlled standard sinusoidal loading and the shear strain level of the loading amplitude is linearly increased from 0.001 to 0.3. Taking the LATS test of the WBP-modified asphalt at 20 °C as an example, the method for determining the *τ*_cri_ is analyzed. The curves of phase angle (*δ*) and shear modulus (G*) as functions of shear strain (*γ*) are shown in Figure 11.

As presented, the loading amplitude is less than the nonlinear viscoelastic critical point corresponding to the nonlinear viscoelastic critical shear strain (*γ*_cri_) and shear stress (*τ*_cri_) with no damage accumulation, resulting in the *δ* and G* remaining in an almost linear and steady state. As the loading level gradually increases, damage accumulates, causing the *δ* to first increase and then decrease, while the G* decreases nonlinearly. The *γ* corresponding to the intersection point where the G* rapidly decreases and the linear steady phase ends is taken as the *γ*_cri_. The shear stress corresponding to the *γ*_cri_ is recorded as the *τ*_cri_, and the obtained *τ*_cri_ of the WBP-modified asphalt at 20 °C is 95 kPa.

#### 4.2.2. Non-Damage Time Sweep (NDTS) Test

**Figure 12:**
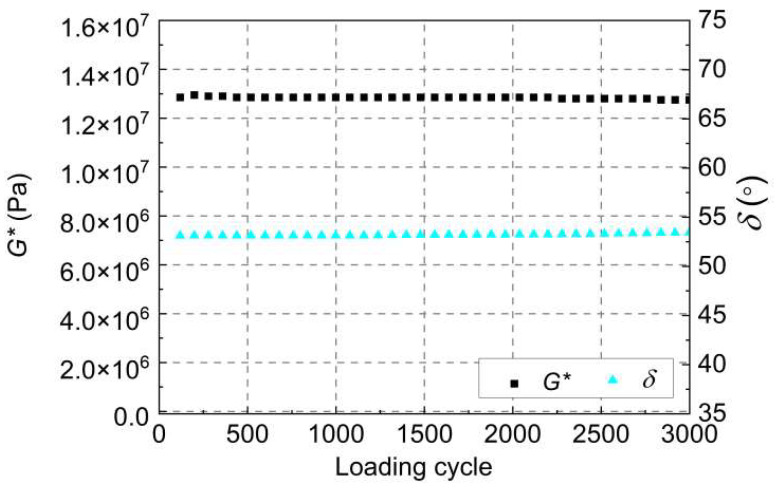
NDTS test results of WBP-modified asphalt.

The stress-controlled NDTS tests were utilized to analyze the non-damaging mechanical response of the tested asphalt. The loading waveform is set as a standard sinusoidal loading. The loading amplitude and loading time are *τ*_cri_ and 300 s, respectively. Figure 12 presents the results of *δ* and G* for the WBP-modified asphalt at 20 °C. As observed, under cyclic loading with an amplitude of *τ*_cri_, the WBP-modified asphalt exhibits *δ* and G* values that do not produce significant changes, which is due to the fact that the WBP-modified asphalt tested by the *τ*_cri_ is in a non-destructive phase.

#### 4.2.3. Damage Time Sweep (DTS) Test

**Figure 13:**
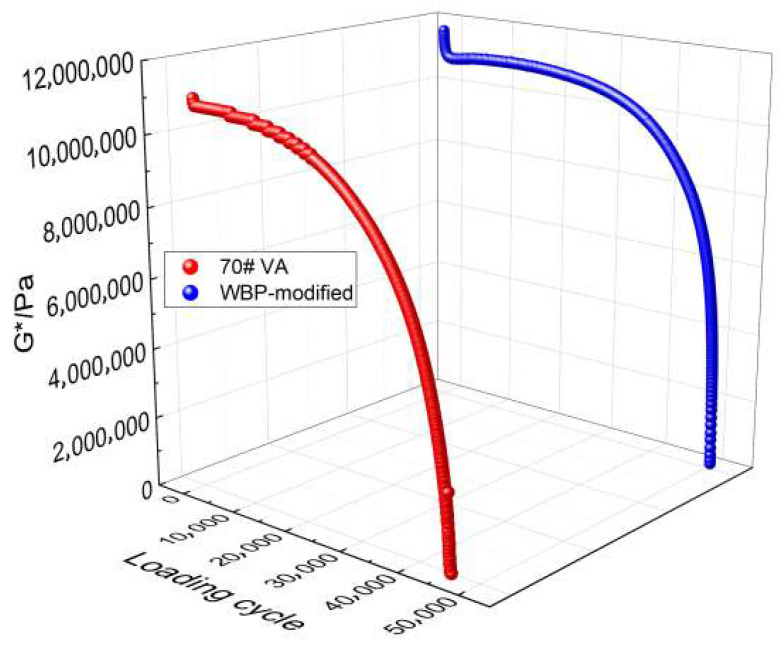
DTS test results of WBP-modified and sol–gel 70# virgin asphalt.

The damage mechanical response of the 70# virgin and the WBP-modified asphalt was analyzed by the DTS tests. First, the asphalt specimen tested by the NDTS test is allowed to recover for 300 s. Then, the stress-controlled standard sinusoidal loading with the amplitude shown in Table 5 is applied to the same asphalt specimen. Taking the tested WBP-modified and sol–gel 70# virgin asphalt under 20 °C and 100 kPa as an analysis example, their G* decay trend is described. Figure 13 shows the corresponding G* results. As can be seen, G* gradually exhibits a nonlinear decay, which is due to the stress amplitude being greater than the *τ*_cri_, resulting in the crack damage induced during cyclic loading. In addition, the G* decay rate of the WBP-modified asphalt is less than that of the sol–gel 70# virgin asphalt, which indicates that the asphalt damage evolution rate can be delayed by the WBP.

#### 4.2.4. Scanning Electron Microscope (SEM) Test

**Figure 14:**
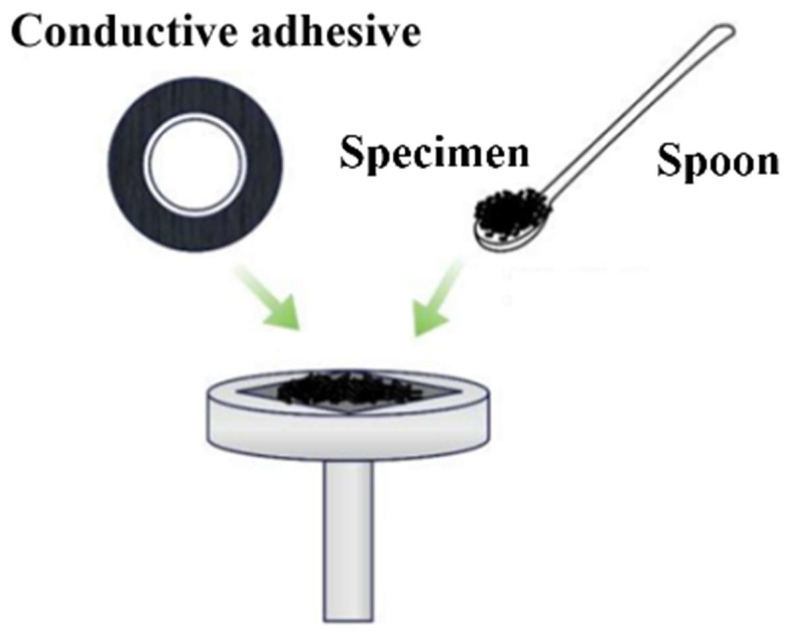
Specimen preparation procedure of the SEM test.

The microstructures of WBP and the WBP-modified asphalt were measured using an IT300 SEM machine and Figure 14 illustrates the corresponding specimen preparation procedure. The SEM selected in this paper was produced by Chinainstru & Quantumtech (Hefei) Co., Ltd. (Hefei, Anhui Province, China). The WBP or asphalt was dried to remove moisture to obtain the test samples. Conductive adhesive was applied to the specimen stage. The WBP or asphalt specimen was placed onto the adhesive using a chemical spoon. The microstructures of WBP and the WBP-modified asphalt were then extracted and analyzed.

## Figures and Tables

**Table 1:** Loading amplitudes and fatigue life of destructive time sweep test.

Asphalt Type	Temperature/°C	Loading Amplitude/kPa	Fatigue Life	Coefficients of Variation/%
Sol–gel 70# virgin asphalt	15	250	79,530	8.736
	20	100	45,140	8.698
	25	80	25,890	9.057
WBP-modified asphalt	15	250	87,880	11.468
	20	100	50,466	10.176
	25	80	29,022	10.797

**Table 2:** Matching results of damage model.

Asphalt	Temperature/°C	*β*	*R* ^2^
WBP-modified asphalt	15	6.14982	0.901
	20	5.38166	0.917
	25	4.40148	0.896
Sol–gel 70# virgin asphalt	15	7.11267	0.902
	20	6.55815	0.901
	25	6.13306	0.897

**Table 3:** Basic parameter indicators of sol–gel 70# virgin asphalt.

Indicators	Test Conditions	Results
Softening point/°C	135 °C	48
Viscosity/(m Pa·s)	-	27
Penetration/(0.1 mm)	25 °C	69

**Table 4:** Physical parameter indicators of WBP.

Technical Indices	Results
Specific surface area/(m^2^·g^−1^)	0.045
Particle/mm	<0.075

**Table 5:** Loading amplitudes of the DTS tests.

Asphalt Type	Temperature/°C	Loading Amplitude/kPa
sol–gel 70# virgin asphalt	15	250
	20	100
	25	80
WBP-modified asphalt	15	250
	20	100
	25	80

## Data Availability

The data are contained within the article. Some or all of the data, models, or code that support the findings of this study are available from the corresponding author upon reasonable request.

## References

[1] Zhang J.W., Chen M.Z., Wu S.P., Chen D.Y., Zhao Y.C., Zhou X.X. (2023). Characteristics of waste dry battery powder and its enhancement effect on the physicochemical properties of asphalt binder. J. Clean. Prod..

[2] Sabbaghi M., Behdad S. (2024). Estimating energy left in discarded alkaline batteries: Evaluating consumption and recovery opportunities. Waste Manag..

[3] Gao W.F., Wang J.Q., Wang Z.Q., Wang Z., Wang Z.L., Cui H., Zeng X.J., Wang G.H., Lv L.Y., Sun Z. (2024). Analysis of the current status of national and local policies on the recycling of used power batteries in China. Chem. Ind. Eng. Prog..

[4] Ruan D.S., Wang F.M., Wu L., Du K., Zhang Z.H., Zou K., Wu X.F., Hu G.R. (2020). A high-performance regenerated graphite extracted from discarded lithium-ion batteries. New J. Chem..

[5] Azam M.G., Kabir M.H., Shaikh M.A.A., Ahmed S., Mahmud M., Yasmin S. (2022). A rapid and efficient adsorptive removal of lead from water using graphene oxide prepared from waste dry cell battery. J. Water Process. Eng..

[6] Roy D., Neogi S., De S. (2021). Adsorptive removal of heavy metals from battery industry effluent using MOF incorporated polymeric beads: A combined experimental and modeling approach. J. Hazard. Mater..

[7] Roy I., Sarkar G., Mondal S., Rana D., Bhattacharyya A., Saha N.R., Adhikari A., Khastgir D., Chattopadhyay S., Chattopadhyay D. (2016). Synthesis and characterization of graphene from waste dry cell battery for electronic applications. RSC Adv..

[8] Liu G.N., Yu Y.J., Hou J., Xue W., Liu X.H., Liu Y.Z., Wang W.H., Alsaedi A., Hayat T., Liu Z.T. (2014). An ecological risk assessment of heavy metal pollution of the agricultural ecosystem near a lead-acid battery factory. Ecol. Indic..

[9] Liu J.W., Hao P.W., Dou Z.S., Wang J.B., Rheological L.J.M. (2021). Healing and microstructural properties of unmodified and crumb rubber modified asphalt incorporated with graphene/carbon black composite. Constr. Build. Mater..

[10] Jiang H., Shao J.A., Zhu Y.J., Yu J., Cheng W., Yang H.P., Zhang X., Chen H.P. (2023). Production mechanism of high-quality carbon black from hightemperature pyrolysis of waste tire. J. Hazard. Mater..

[11] Feng X.J., Zha X.D., Cheng J. (2012). Preparation and properties of PAN-based carbon fiber conductive asphalt concrete. China J. Highw. Transp..

[12] He J., Hu W., Xiao R., Wang Y.H., Polaczyk P., Huang B.S. (2022). A review on Graphene/GNPs/GO modified asphalt. Constr. Build. Mater..

[13] Guo F., Li X.Y., Wang Z.R., Chen Y.J., Yue J.C. (2024). Study on the Factors Affecting the Self-Healing Performance of Graphene-Modified Asphalt Based on Molecular Dynamics Simulation. Polymers.

[14] Liu Z.Q., Liang K., Cao X.J., Chen J.C., Ji S.Z., Shen Q.R. (2024). Influence of graphene on the composite modified asphalt based on viscoelastic and elastic properties. Constr. Build. Mater..

[15] Nie F.H., Chow C.L., Lau D. (2023). Effect of functionalization and defects on thermal conductivity of graphene sheets modified asphalt nanocomposites. Appl. Surf. Sci..

[16] Liu S.T., Cao W.D., Shang S.J., Qi H., Fang J.G. (2010). Analysis and application of relationships between low-temperature rheological performance parameters of asphalt binders. Constr. Build. Mater..

[17] Hu X.F., Robles A., Vikstrom T. (2021). A novel process on the recovery of zinc and manganese from spent alkaline and zinc-carbon batteries. J. Hazard. Mater..

[18] Zhu H.R., Yu M.M., Zhang Y., Wang W.F. (2022). Study on wave-absorbing and warming properties of asphalt mixtures doped with magnetite powder/manganese dioxide. Highw. Eng..

[19] Zhao D., Jiang J., Gu X., Liu J., Wang J., Yang G. (2025). Value-added recycling of plant waste for modification of asphalt pavement used aggregates: Interface enhancement and carbon sequestration. Chem. Eng. J..

[20] Liu T.C., Jiang J.W., Lu G.Y., Ding J.T., Ni F.J. (2026). Adsorption-state test guided enhancement of asphalt emulsion–aggregate interface: From microscopic defects to macroscopic strengthening. Constr. Build. Mater..

[21] Liu K.F., Zhang K., Shi X.M. (2018). Performance evaluation and modification mechanism analysis of asphalt binders modified by graphene oxide. Constr. Build. Mater..

[22] Jahanbakhsh H., Karimi M.M., Jahangiri B.F., Moghadas N. (2018). Induction heating and healing of carbon black modified asphalt concrete under microwave radiation. Constr. Build. Mater..

[23] Liu Z.M., Yang X., Wang Y.D., Luo S. (2019). Engineering properties and microwave heating induced ice-melting performance of asphalt mixture with activated carbon powder filler. Constr. Build. Mater..

[24] Fan L.P. (2023). Evaluation of the Effect of Asphalt Modified by Waste Batteries, Micro-Mechanism and its Mixture Properties.

[25] Meng Y.J., Chen P.Y., Fan L.P., Rong H.L., Lai J., Zhang C.Y., Chen J.P., Li Y.W. (2024). Investigation into the modification mechanism and performance assessment of waste battery powder modified bitumen. Int. J. Pavement Eng..

[26] Meng Y.J., Lai J., Fan L.P., Mo S.Y., Guo C.L., Zhang C.Y. (2023). Recycling of the waste battery: Effect of waste battery on property of asphalt and environmental impact evaluation. Sci. Total. Environ..

[27] Meng Y.J., Fan L.P., Chen J., Liao Y.J., Han H.H. (2021). Microscopic characterization of waste battery powder modified bitumen and its performance. China J. Highw. Transp..

[28] Gan X.L., Chen P., Yu B., Zhang W.G. (2023). Study on the performances of waste battery powder modified asphalt and asphalt mixture. Polymers.

[29] Fang C.Z., Guo N.S., Li H., Leng Z., Jiang J.W. (2024). Investigating fatigue damage accumulation of asphalt binders considering amplitude sequence and loading interaction. J. Mater. Civ. Eng..

[30] Li H., Luo X., Yan W., Zhang Y.Q. (2020). Pseudo energy-based kinetic characterization of fatigue in asphalt binders. China J. Highw. Transp..

[31] Lv S.T., Liu C.C., Zheng J.L., You Z.P., You L.Y. (2018). Viscoelastic Fatigue Damage Properties of Asphalt Mixture with Different Aging Degrees. KSCE J. Civ. Eng..

[32] Zheng J.L., Lv S.T., Tian X.G. (2010). Research on viscoelastic fatigue damage model of aging asphalt mixture. Eng. Mech..

[33] Lv S.T., Liu C.C., Lan J.T., Zhang H.W., Zheng J.L., You Z.P. (2018). Fatigue equation of cement-treated aggregate base Materials under a true stress ratio. Appl. Sci..

[34] Lv S.T., Zhao T.D., Xia C.D., Zhao S.G., Liu T.J., Liu Y.H., Liu B., Cabrera M.B. (2022). A new method for characterizing the fatigue performance of high-modulus asphalt mixtures. J. Test. Eval..

[35] Li H., Luo X., Zhang Y.Q. (2021). A kinetics-based model of fatigue crack growth rate in bituminous material. Int. J. Fatigue.

[36] Fang C.Z., Tang D.Q., Li Z.X., Chen Y.Z., Guo N.S., Guo T.T. (2024). A new fatigue equation for asphalt mixtures considering loading sequence effects. Case Stud. Constr. Mater..

[37] Fang C.Z., Li H., Li Z.X., Chen Y.Z., Guo T.T. (2025). Fatigue life prediction of asphalt binders under variable loading levels based on dissipated pseudo-strain energy. Case Stud. Constr. Mater..

